# Intraoperative imaging of folate receptor alpha positive ovarian and breast cancer using the tumor specific agent EC17

**DOI:** 10.18632/oncotarget.8282

**Published:** 2016-03-22

**Authors:** Quirijn R.J.G. Tummers, Charlotte E.S. Hoogstins, Katja N. Gaarenstroom, Cor D. de Kroon, Mariette I.E. van Poelgeest, Jaap Vuyk, Tjalling Bosse, Vincent T.H.B.M Smit, Cornelis J.H. van de Velde, Adam F. Cohen, Philip S. Low, Jacobus Burggraaf, Alexander L. Vahrmeijer

**Affiliations:** ^1^ Department of Surgery, Leiden University Medical Center, Leiden, The Netherlands; ^2^ Department of Gynecology, Leiden University Medical Center, Leiden, The Netherlands; ^3^ Department of Anesthesiology, Leiden University Medical Center, Leiden, The Netherlands; ^4^ Department of Pathology, Leiden University Medical Center, Leiden, The Netherlands; ^5^ Centre for Human Drug Research, Leiden, The Netherlands; ^6^ Department of Chemistry, Purdue University, West Lafayette, IN, USA

**Keywords:** image-guided surgery, fluorescence, folate-receptor alpha, breast cancer, ovarian cancer

## Abstract

**Introduction:**

Intraoperative fluorescence imaging of the folate-receptor alpha (FRα) could support completeness of resection in cancer surgery. Feasibility of EC17, a FRα-targeting agent that fluoresces at 500nm, was demonstrated in a limited series of ovarian cancer patients. Our objective was to evaluate EC17 in a larger group of ovarian cancer patients. In addition, we assessed the feasibility of EC17 in patients with breast cancer.

**Methods:**

Two-to-three hours before surgery 0.1mg/kg EC17 was intravenously administered to 12 patients undergoing surgery for ovarian cancer and to 3 patients undergoing surgery for biopsy-proven FRα-positive breast cancer. The number of lesions/positive margins detected with fluorescence and concordance between fluorescence and tumor- and FRα-status was assessed in addition to safety and pharmacokinetics.

**Results:**

Fluorescence imaging in ovarian cancer patients allowed detection of 57 lesions of which 44 (77%) appeared malignant on histopathology. Seven out of these 44 (16%) were not detected with inspection/palpation. Histopathology demonstrated concordance between fluorescence and FRα- and tumor status. Fluorescence imaging in breast cancer patients, allowed detection of tumor-specific fluorescence signal. At the 500nm wavelength, autofluorescence of normal breast tissue was present to such extent that it interfered with tumor identification.

**Conclusions:**

FRα is a favorable target for fluorescence-guided surgery as EC17 produced a clear fluorescent signal in ovarian and breast cancer tissue. This resulted in resection of ovarian cancer lesions that were otherwise not detected. Notwithstanding, autofluorescence caused false-positive lesions in ovarian cancer and difficulty in discriminating breast cancer-specific fluorescence from background signal. Optimization of the 500nm fluorophore, will minimize autofluorescence and further improve intraoperative tumor detection.

## INTRODUCTION

Over the past decades multiple imaging modalities have become available for preoperative detection of tumors, staging disease and identifying sentinel lymph nodes [1, 2]. However, translation of preoperative obtained images to the surgical theatre can be challenging. Consequently, surgeons largely have to rely on visual inspection and palpation to discriminate between healthy and malignant tissue. As a result, incomplete resection of malignant tissue may occur. In breast cancer surgery, for example, positive resection margins are reported in up to 20% of patients after resection of the primary tumor [3]. In metastasized disease, intraoperative imaging of tumor tissue can be of great advantage. In ovarian cancer for example, clear intraoperative detection of metastatic lesions can improve staging procedures in early stage ovarian cancer (FIGO I and IIa), and facilitate complete or optimal cytoreductive surgery in advanced stage disease (FIGO IIb to IV). Both the prevention of positive margins in solid tumors and the performance of adequate staging and complete/optimal cytoreduction will improve individual patient outcome [4–9]. Hence there is a clear unmet need for intraoperative modalities that can identify tumor tissue with high sensitivity and specificity.

An innovative intraoperative optical imaging technique is fluorescence imaging. Over the past years multiple studies have been performed on tumor imaging, sentinel lymph node (SLN) mapping and identification of vital structures, using fluorescence imaging [10, 11].

Optical properties of fluorescent contrast agents are of importance for successful tumor imaging. The wavelength of the fluorescent light largely determines the degree of penetration of photons into the tissue. Photons in the visible light range have a depth penetration limited to a few millimeters and are suitable for detection of superficial targets. Conversely photons in the NIR range (650-900 nm) can travel more than a centimeter through tissue, which also enables detection of targets below the tissue surface [12]. Moreover, the wavelength of the fluorescent light also plays a role in autofluorescence. Autofluorescence is fluorescence arising from intrinsic tissue components after excitation with UV, visible, or NIR radiation of suitable wavelength. To detect cancer cells targeted with an optical contrast agent, the signal of the target-specific fluorescence must be significantly higher than the autofluorescence. The occurrence of autofluorescence is determined by the tissue type and excitation wavelength [13, 14].

Biological characteristics of fluorescent contrast agents are essential to achieve target-specific fluorescence imaging. Ideally, a contrast agent binds exclusively to a cancer specific ligand, while being excreted rapidly from the rest of the body. Over the past years, extensive preclinical validation of tumor-specific contrast agents targeting a variety of ligands was reported, however only very few were clinically introduced [15, 16]. For this reason, several clinical studies have been performed to explore feasibility of clinically available fluorescent contrast agents like methylene blue (MB) and indocyanine green (ICG) for intraoperative tumor imaging of breast cancer tissue and ovarian cancer tissue [17, 18]. These agents do not specifically bind to the tumor, but make use of other mechanisms such as the Enhanced Permeability and Retention (EPR) [19, 20] effect and disturbed excretion profiles causing accumulation in or around tumor tissue. Although promising results were described, resection of non-malignant lesions due to false positive fluorescence proved an insurmountable problem in the road to clinical application. Consequently, the need for newly developed contrast agents with highly specific binding to tumor-specific targets remains.

A promising target for image-guided surgery is the folate receptor alpha (FRα). Normally the FRα is expressed only at low levels and due to its location on the apical membrane of epithelial cells it is not accessible for molecules transported by blood [21, 22]. In contrast, in many types of epithelial cancers, the FRα is highly expressed. As a result of the loss of cell polarity in cancer, the FRα is easily accessible by blood making it an ideal tumor target. Over 90% of all epithelial ovarian cancers over-express FRα, and in ovarian cancers of serous morphology this percentage is even higher (90-100%) [23–25]. Moreover, expression is not altered by chemotherapy [26, 27], allowing use of this target in both primary and interval cytoreductive surgical procedures. In breast cancer, FRα overexpression is reported in 30% of tumors, this percentage is even higher (67%) in tumors with a “triple-negative” receptor profile [28]. In almost all breast cancer patients, preoperative biopsies are available, allowing characterization of FRα status before surgery to select patients who will benefit from FRα targeted imaging agents.

While multiple preclinical studies have been performed on the imaging of FRα positive tumors [29–31], clinical experience is very limited. Van Dam et al. showed feasibility of intraoperative imaging of ovarian cancer metastases using EC17, a FRα targeting contrast agent with fluorescent properties in the visible light spectrum (500nm). In a limited series of patients undergoing surgery for suspected ovarian cancer, fluorescent tumor tissue was observed intraoperatively after intravenous administration of EC17 in 3 out of 4 patients with proven ovarian cancer. However, the intra-operative detection of additional tumor lesions due to the use of EC17 and fluorescence imaging was not reported. Moreover, administration of the contrast agents had to be interrupted, although the study could be completed, in several (4 out of 10) patients due to mild adverse events. As a result the initial dose of 0.3 mg/kg was decreased to 0.1 mg/kg, which reduced adverse events while maintaining fluorescent signal. To demonstrate the tolerability and additional value of fluorescence imaging using EC17 in ovarian cancer, evaluation in a larger patient cohort is required. Although the overexpression of the FRα on selected breast cancer cells has been shown, feasibility of fluorescence imaging using EC17 in patients with FRα positive breast cancer has not yet been demonstrated.

Our objective was to evaluate a relatively low dose of EC17 (0.1 mg/kg) in a larger group of ovarian cancer patients and to assess feasibility of intraoperative fluorescence imaging in patients with FRα positive breast cancer.

## RESULTS

### Patient characteristics

A total of 13 ovarian cancer patients were included. Surgery was cancelled in 1 patient due to deterioration of the medical condition of the patient prior to surgery. Thus 12 ovarian cancer patients received EC17 and underwent open surgery; a tabular overview of the patient characteristics, FIGO status and histology of the tumor type is given in Table 1. Six patients underwent a primary cytoreductive procedure, 4 patients an interval cytoreductive procedure and 2 patients a staging procedure.

**Table 1:** Demographic and baseline characteristics of ovarian cancer patients

Patient ID	Age	Surgical procedure	Diagnosis	FIGO stage	Metastases identified	Tumor FRa+	Fluorescence imaging successful
1	71	Primary debulking	Serous adenocarcinoma	3c	Yes	Yes	Yes
2	51	Primary debulking	Endometroid type adenocarcinoma	3b	Yes	Yes	Yes
3	59	Staging	Endometroid type adenocarcinoma	2c	No	Yes	Yes
4	61	Interval debulking	Serous adenocarcinoma of endometrium	4	Yes	Yes	Yes
5	64	Primary debulking	Borderline serous adenocarcinoma	3b	Non-invasive implants	Yes	Yes
6	71	Primary debulking	Serous adenocarcinoma	3c	Yes	Yes	Yes
7	71	Primary debulking	Serous adenocarcinoma	3b	Yes	Yes	Yes
8	78	Interval debulking	Serous adenocarcinoma	3c	Yes	Yes	Yes
9	57	Interval debulking	Serous adenocarcinoma	3c	Yes	Yes	Yes
10	52	Interval debulking	Serous adenocarcinoma	4	Yes	Yes	Yes
11	42	Primary debulking	Mucinous adenocarcinoma	3c	Yes	No	No
12	73	Staging	Endometroid type adenocarcinoma	2b	No	Yes	Yes

For breast cancer, a total of 53 potentially eligible breast cancer patients were selected for characterization of FRα status on preoperatively obtained biopsies. Samples of six patients stained positive for FRα (11%). Of the 6 patients, 2 patients eventually did not meet the inclusion criteria and 1 patient declined participation. Three patients were included in the study and their characteristics are provided in Table 2.

**Table 2:** Demographic and baseline characteristics of breast cancer patients

Patient ID	Age	Surgical procedure	Diagnosis	Tumor size in mm	ER status	PR Status	HER2 Status	SLN metastasis	Tumor FRa+	Fluorescence imaging successful
1	54	BCS	IBC NST	19	Neg	Neg	Neg	Yes, 9mm	Yes	Yes*
2	61	BCS	Metaplastic carcinoma	42	Neg	Neg	Neg	No	Yes	Yes
3	53	Mastectomy	IBC NST	23	Pos	Pos	Neg	No	Yes	Yes

Abbreviations: BCS, Breast Conserving Surgery; IBC, infiltrative breast cancer; NST, No Special Type

* Tumor successfully identified in resection specimen. SLN metastasis not identified due to lack of tissue penetration.

### Safety

All patients received 0.1 mg/kg EC17 over 10 minutes, and no infusion was intermitted or stopped. Infusion of EC17 was associated with mild, self-limiting hypersensitivity reactions in 7 out of 15 patients. The symptoms consisted of abdominal discomfort, itching throat and sneezing (for a summary list of treatment related adverse events, see Supplementary Table S1). One patient vomited after EC17 administration and received ondansetron 8 mg intravenously, followed by the planned surgical procedure. There were no clinical relevant changes in blood pressure or pulse rate compared to baseline.

### Intraoperative fluorescence imaging

**Figure 1:**
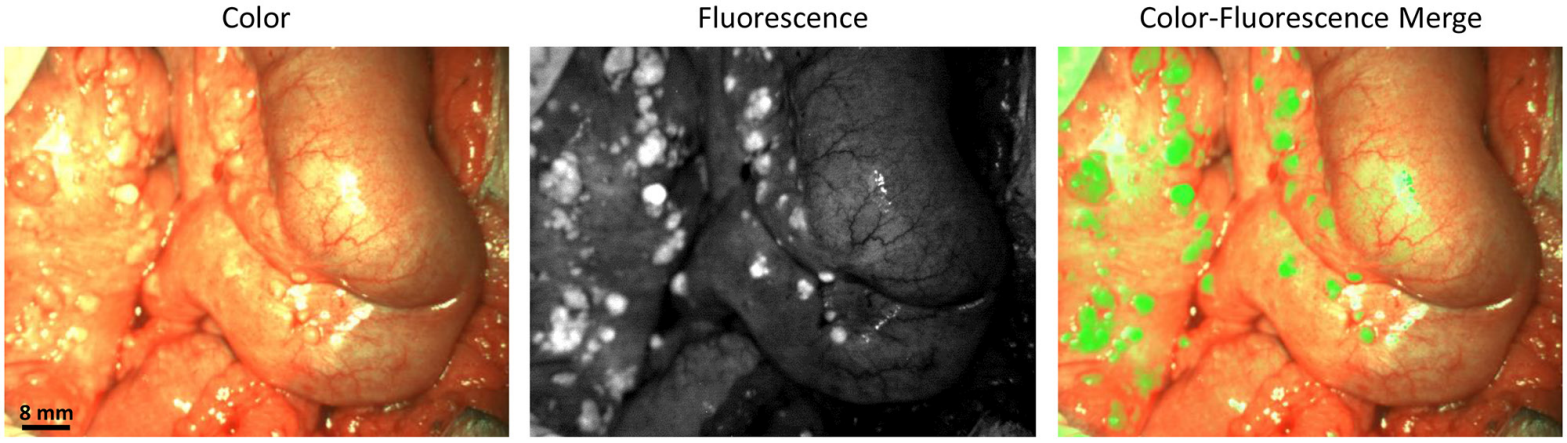
Identification of ovarian cancer metastases using fluorescence imaging Identification of ovarian cancer metastases located on the intestine and mesentery using fluorescence imaging. Biopsies of lesions were found histologically to be metastases of serous adenocarcinoma.

Intraoperative fluorescence imaging in ovarian cancer patients allowed clear detection of ovarian cancer lesions. Figure 1 shows an example of fluorescent ovarian cancer metastases. In total, 57 fluorescent lesions that were identified during surgery were resected. Of these resected lesions 44 (77%) appeared to be malignant on histopathology. Seven (16%) of these 44 lesions were not detected by visual inspection with the naked eye or palpation either because they appeared benign or because they were missed during inspection due to small size (<10mm) and flat nature. These lesions were only removed because these could be identified using fluorescence imaging. Mean TBR was 7.0 ± 1.2. Fluorescence imaging was successful up to about 5.5 hours after EC17 administration, which was the longest time interval measured between administration and the end of a surgical procedure.

**Figure 2:**
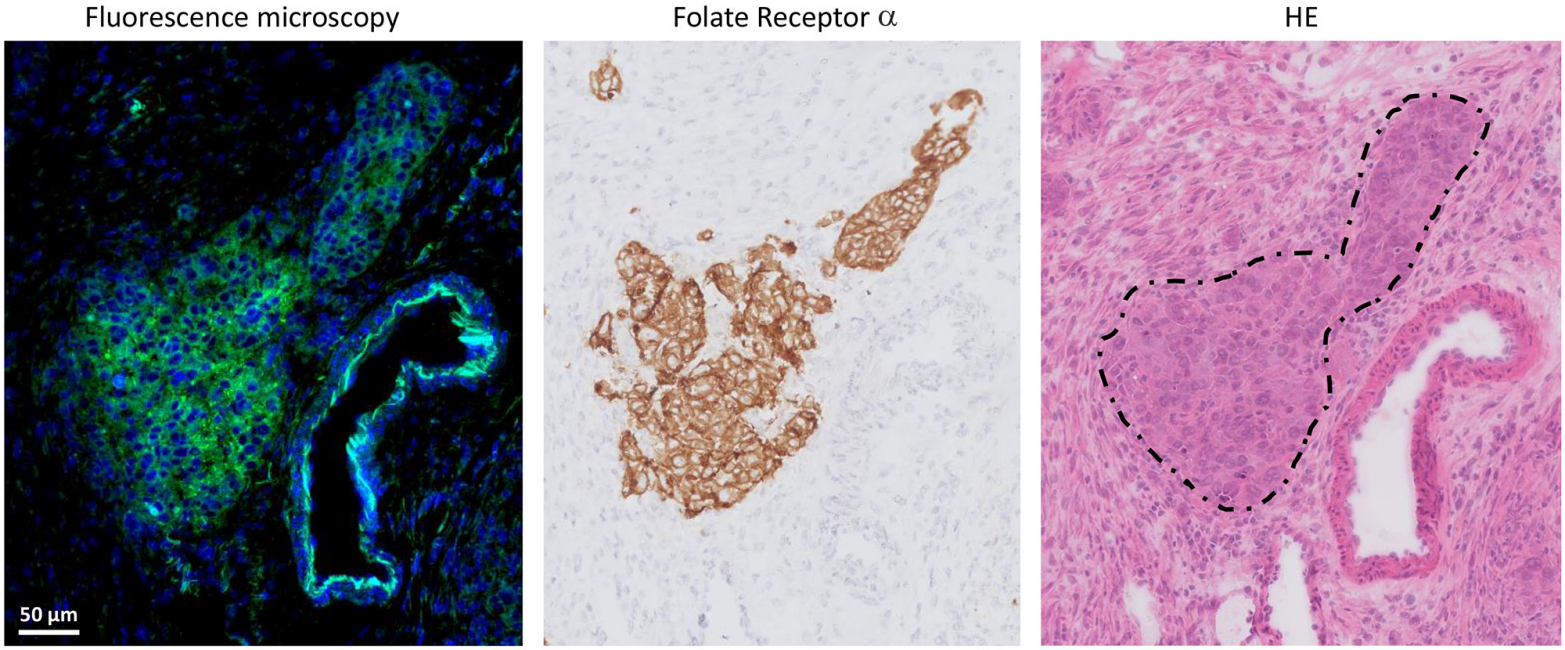
Histopathological evaluation of fluorescence signal in ovarian cancer Fluorescence signal is indicated with green, blue color represents cell nuclei stained with DAPI. Fluorescence microscopy showed clear membranous and cytoplasmic accumulation of EC17 in tumor cells. The fluorescent signal is located on all sites that stain positive for FRα expression, which is the anatomical site that appears to be a metastasis of serous ovarian adenocarcinoma on hematoxylin and eosin staining (dashed circle).

Histopathology demonstrated clear concordance between fluorescence and FRα- and tumor status. Fluorescence microscopy showed clear membranous and cytoplasmic accumulation of EC17 in tumor cells (Figure 2).

The (ex-vivo) assessment of the stills obtained from the videos made during the surgical procedure showed that on average (SD) 23.3 (± 11.9) lesions per still were identified with the naked eye. When the stills were supplemented with the fluorescence image, 39.6 (± 22.7) lesions per still were identified, a 70% increase.

**Figure 3:**
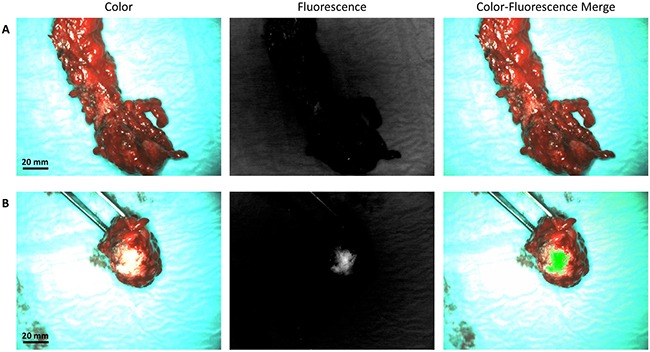
False-negative fluorescent signal caused by a lack of depth penetration **A.** Example of a metastasized greater omentum, which was clinical suspicious, but showed no fluorescence signal from the outside. **B.** After dissecting the omentum, strong fluorescent signal was identified. This observation shows the lack of tissue penetration at 500nm.

In total, 3 false-negative lesions were identified. These lesions were all metastases in the greater omentum. The lesions were considered suspicious for malignancy by the surgeons, but showed no intraoperative fluorescence signal from the outside (Figure 3A). However, upon dissection of the omentum on the backtable, strong fluorescent signal was identified (Figure 3B), suggesting that the intraoperative non-fluorescence was caused by the lack of tissue penetration at 500nm wavelength.

Thirteen out of the 57 (23%) fluorescent lesions appeared benign. Five of these false positive lesions were identified as normal fallopian tube tissue on histopathological evaluation, showing FRα expression. These lesions were thus expected to bind EC17, and were resected anyway as part of the standard surgical procedure. Six lesions were structures mainly containing collagen, which is known to cause autofluorescence at 500nm. Mean TBR of the false-positives was 5.4 ± 1.0. There was no significant difference in TBR between true-positives and false-positives (7.0 vs. 5.4; P = 0.47). Characteristics of false positive and false negative lesions are provided in Table 3.

**Table 3:** Characteristics of false positive and false negative fluorescent lesions in ovarian cancer

Patient ID	Location lesion	False positive or negative	Probable cause
1	Fallopian tube	False positive	FRα expression
2	Iliacal lymph node	False positive	FRβ expression activated macrophages
2	Ligamentum rotundum	False positive	Autofluorescence collagen containing structure
4	Omentum	False negative	Inadequate penetration depth
5	Leiomyoma Uterus	False positive	Autofluorescence collagen containing structure
5	Leiomyoma Uterus	False positive	Autofluorescence collagen containing structure
5	Omentum biopsy	False positive	Unknown
7	Omentum	False negative	Inadequate penetration depth
8	Omentum	False negative	Inadequate penetration depth
8	Fallopian tube	False positive	FRα expression
10	Fallopian tube	False positive	FRα expression
12	Cervix	False positive	Autofluorescence collagen containing structure
12	Myometrium uterus	False positive	Autofluorescence collagen containing structure
12	Fallopian tube	False positive	FRα expression
12	Infundibulopelvic ligament	False positive	Autofluorescence collagen containing structure
12	Ovary (contralateral)	False positive	FRα expression

**Figure 4:**
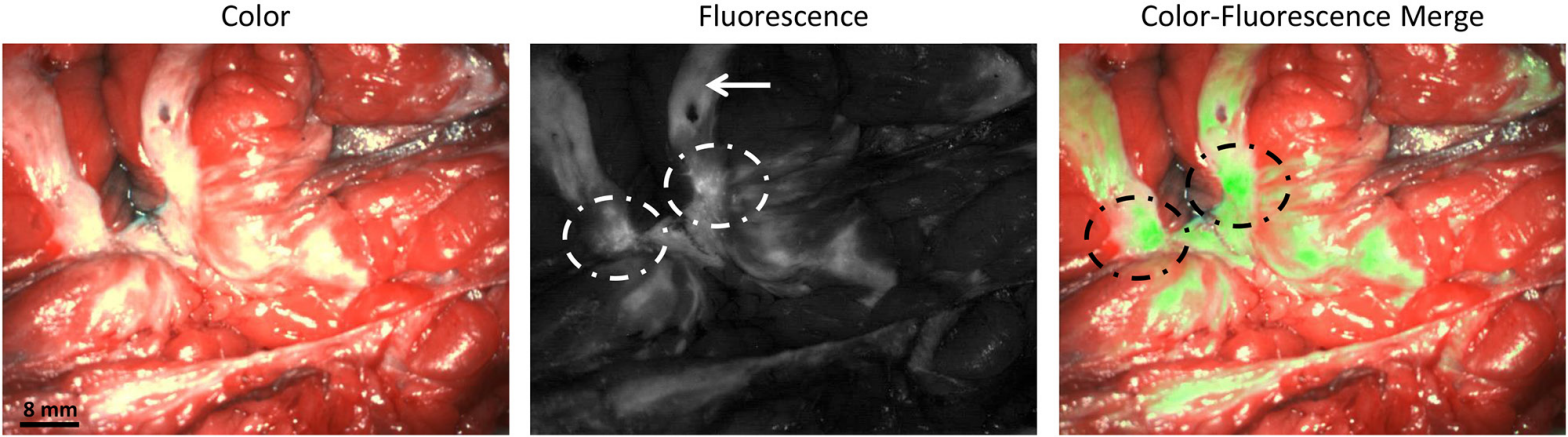
Identification of breast cancer metastases using fluorescence imaging Identification of a bisected primary breast cancer lesion using fluorescence imaging (dashed circles). The arrow indicated autofluorescence signal from normal breast tissue. The tumor was found histologically to be an infiltrating breast cancer of no special type.

**Figure 5:**
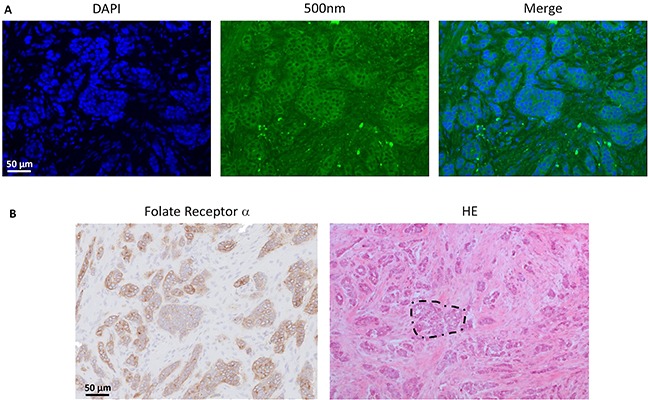
Histopathological evaluation of fluorescence signal in breast cancer **A.** Fluorescence microscopy showed clear membranous and cytoplasmic accumulation of EC17 in tumor cells. Blue color represents cell nuclei stained with DAPI, fluorescence signal is indicated with green. Also a relatively high diffusely fluorescent background signal is seen, which is in concordance with the fluorescence images obtained with the intraoperative imaging system. **B.** Immunohistological staining for FRα expression shows a FRα positive infiltrating breast cancer of no special type (example in dashed circle). Clear concordance is observed between fluorescent signal and FRα positive malignant lesions.

Also in breast cancer, tumor-specific fluorescence signal was observed. Median TBR was 2.3 (range 2.1 – 6.2). However, autofluorescence of normal breast tissue was present to such extent that it interfered with tumor identification. Figure 4 shows fluorescent signal in breast cancer tissue and in normal, autofluorescent breast tissue. Fluorescence microscopy showed clear membranous and cytoplasmic accumulation of EC17 in tumor cells (Figure 5). In addition, high background fluorescent signal was observed, which is in concordance with images obtained with the intraoperative Artemis imaging system.

In one patient with breast cancer, a tumor-positive SLN was found on histopathological evaluation. This SLN however was not detected with fluorescence imaging. After HE and FRα staining, the metastasis appeared to be in the center of the SLN, and was therefore not detected due to lack of tissue penetration.

### Pharmacokinetics

The maximal concentration for each dose was obtained directly after the end of the infusion and declined thereafter with a half-life of 86.8 minutes.

## DISCUSSION

Intraoperative imaging of tumor tissue may improve patient outcome by enhanced identification and subsequent resection of tumor tissue. Fluorescence guided surgery using the FRα specific contrast agents EC17 allowed real time identification of both ovarian- and breast cancer cells. In ovarian cancer, the intraoperative use of fluorescence imaging resulted in the resection of 16% more malignant lesions compared to inspection with the naked eye and/or palpation only. Visual identification was improved on stills made from intraoperative videos, supporting the notion that fluorescent imaging improves detection even when other techniques like palpation are not available.

The biophysical properties of imaging agents are of paramount importance for successful tumor identification. Ideally, high fluorescence signal is observed in malignant lesions, while normal or healthy tissue shows minimal fluorescence because of a low binding constant and fast excretion of the imaging agent after initial biodistribution. The size of the compound greatly influences this profile. Currently monoclonal antibodies, antibody fragments, such as single-chain (scFv) of fab fragments, small peptides or structure-inherent targeting fluorophores are used for tumor-specific imaging [32–36]. The biodistribution, excretion and binding profile of EC17 shows several great advantages in imaging. The maximum concentration (Cmax) of EC17 is observed directly after the end of administration and is followed by a rapid excretion from the blood. While fluorescence signal of the tumor is observed up to more than 5 hours after administration of the compound. This short terminal half-life in blood, strong tumor specific signal and low background signal allows tumor imaging from 2 hours post dosing and during a relative long time.

Around 75% of patients with ovarian cancer present with advanced stage [37] of the disease. Multiple studies have shown that the amount of residual disease is the most important prognostic factor for survival in ovarian cancer patients. As a result of these studies, consensus exists that all attempts should be made to achieve complete cytoreduction i.e. complete removal of all macroscopically visible tumor tissue. [4, 6, 8, 38, 39]. In this study, real-time visualization of malignant lesions using fluorescence imaging during surgery led to the detection of 16% additional malignant lesions. This may improve cytoreduction and hereby patient outcome. The effect of the addition of intraoperative fluorescence imaging on survival was already shown by Stummer et al. in patients with brain glioma. They demonstrated that fluorescence imaging with 5-ala not only leads to more complete resections but also to improved progression free survival [5]. For ovarian cancer more prospective research is necessary to establish the effect on overall survival.

When patients present with clinically early stage ovarian cancer, a staging procedure is recommended. During this procedure, biopsies of suspicious lesions are taken, supplemented with “blind” biopsies from predefined locations. Ultimate goal is to identify metastatic lesions whenever present in order to give adequate treatment i.e. systemic chemotherapy. Visualizing metastatic lesions by fluorescence imaging may optimize staging procedures, as less “blind” biopsies have to be taken. This could facilitate discrimination between true early stage ovarian cancer and more advanced stage with occult tumor spread. Especially in minimal-invasive surgery, when tactile information (palpation) of lesions cannot be obtained, fluorescence imaging could be of additional value.

In breast cancer, up to 20% of patients have positive resection margins after resection of the primary tumor [3]. Visualizing tumor cells during surgery could lower the risk of an incomplete resection as identification of a positive margin can result in direct resection of residual tumor tissue. Although this will probably not influence overall survival, as patient with positive resection margins are currently treated with a re-resection or more intensive radiotherapy, significantly lower healthcare costs and burden to the patient could result. To investigate this concept, both FRα positive breast cancer patients treated with BCS and breast ablation were included in the current feasibility study. However, for future applicability of fluorescence imaging in breast cancer surgery, the most added value is to be expected in BCS. Moreover, a significant number of patients with breast carcinoma is pre-treated with neoadjuvant systemic chemotherapy to reduce the primary tumor to facilitate BCS instead of radical mastectomy [40]. Although pre-treatment with systemic therapy does not result in an increased number of positive resection margins [41], recognition of vital tumor tissue can be challenging. As FRα status is not changed by chemotherapy [27, 42], fluorescence imaging could be of added value in these challenging cases.

In literature, FRα positive breast cancer lesions are described in up to 30% of patient [43–45]. In our series however, only 11% of the obtained biopsies stained positive for FRα. No explanation was found for this lower expression level. A high number of normal breast tissue stained weak positive for FRα, mainly located at the apical surface of epithelial cells and at myoepithelial cells. This finding has been described previously [43, 45], and does not necessarily cause a pitfall for FRα as target for fluorescence guided surgery, because the myoepithelium is not accessible for blood carried contrast agents.

Several limitations of the described technique and contrast agent were caused by the optical properties of the contrast agent. EC17 fluoresces at 488nm, which does not allow identification of lesions located beneath the surface. In 3 patients with ovarian cancer, the greater omentum was suspected for malignancy, but only showed fluorescence after dissection of the tissue and this clearly showed the low tissue penetration of the photons emitted by EC17. We identified 23% false-positive lesions in the patients with ovarian cancer. The non-malignant lesions that fluoresced in this study were in particular collagen-containing structures, from which it is known that they can show autofluorescence in the visible light spectrum [14, 46].

All the above-mentioned limitations could be overcome by conjugating the folate analog to a fluorophore that fluoresces in the NIR spectrum. This allows identification of structures located deeper beneath the surface due to a lower absorption coefficient, and causes less autofluorescence of normal tissue [47]. For surface detection of malignant cells, as in breast cancer, depth penetrating is less important, but currently the autofluorescent signal prevented clinical decision-making. Figure S1 shows fluorescence imaging at 500nm and 800nm of breast tissue containing a tumor. These tissue specimens, from patients that were not treated with an exogenous contrast agent as EC17, are thus suitable to demonstrate background autofluorescence. At 500nm high background autofluorescence is observed, while at 800nm no background autofluorescence signal is seen (Supplementary Methods). This illustrates the need for tumor-specific contrast agents in the NIR spectrum. Currently, our research group is performing a first-in-human clinical trial in ovarian cancer patients using the FRα specific near-infrared contrast agent OTL38. Preclinical tests comparing OTL38 with EC17 have demonstrated superiority of OTL38 in sensitivity and brightness [48].

In conclusion, administration of EC17 was reasonably well-tolerated and produced clear fluorescent signals in ovarian and breast cancer tissue. This allowed resection of 16% more ovarian cancer lesions. Notwithstanding, autofluorescence of benign, predominantly collagen-containing tissues led to detection of a significant proportion of false positive lesions in ovarian cancer. Further, autofluorescence resulted in difficulty in discriminating breast cancer tissue specific fluorescence from background fluorescence. We conclude that FRα is a favorable tumor-specific target, but EC17 lacks the full set of requirements for fluorescence-guided surgery in FRα-positive ovarian and breast cancer, especially because of autofluorescence and insufficient penetration depth. Replacing the 500nm fluorophore by a fluorophore in the NIR spectrum could likely further improve optical properties and thereby clinical relevance of fluorescence-guided surgery.

## MATERIALS AND METHODS

### Investigational agent

EC17 (molecular formula: C42H36N10Na2O10S; On Target Laboratories LLC, West Lafayette, USA) consists of a folate analogue conjugated to 5-fluorescein isothiocyanate (FITC), which is exited between the wavelengths of 465 and 490nm and fluoresces at wavelengths of 520-530 nm. Before administration, the frozen vials containing 5 mg/mL EC17 in 3 mL water for injection were thawed and diluted in 10 mL sterile saline. Patients received 0.1 mg/kg EC17 intravenously over 10 minutes, 2-3 hours before surgery.

### Patients

Patients suspected of early stage epithelial ovarian cancer presenting at the department of Gynecology of the Leiden University Medical Center (LUMC) between February 2014 and September 2014 scheduled to undergo staging surgery or of advanced epithelial ovarian cancer scheduled to undergo cytoreductive surgery, were included in this study. All patients gave written informed consent. For breast cancer, patients presenting at the department of Surgery of the LUMC between May 2014 and February 2015 planned for, either breast conserving surgery (BCS) or breast ablation, were eligible for participation. After selection, preoperatively obtained biopsies of potentially eligible patients were stained for FRα expression using immunohistochemistry (IHC). FRα expression was assessed by using a membranous scoring method with a scale ranging from 0 to 3+, as described by O'shannessy et al. [49] A score of 0 corresponded to absence of staining; 1+ equaled faint staining on luminal borders; 2+ equaled moderate staining on apical and sometimes lateral borders and 3+ indicated strong circumferential staining. The tumor was considered positive when more than 10% of malignant cells were positively stained (>0). Assessment of the stained biopsies was performed by a pathologist (VTHBMS or TB), and after presence of FRα positive tumor cells was confirmed, patients were eligible for EC17 administration. All patients gave written informed consent.

Exclusion criteria were age<18, pregnancy (excluded by pregnancy test in woman of childbearing potential), renal impairment (defined as eGFR<50 mL/min/1.73m^2^), impaired liver function (defined as evidenced by greater than 3x the upper limit of normal (ULN) for ALT, AST, or total bilirubin), or a history of anaphylactic reaction to EC17, insect bites or fluorescein.

### Clinical trial

The study was approved by the Medical Ethics Committee of the LUMC and was performed in accordance with the laws and regulations of the Netherlands. Suitability of selected patients was further assessed by a medical screening consisting of a medical history, physical examination including vital signs, weight, 12-lead ECG, and routine laboratory assessments.

Before administration of EC17, two iv cannulas were inserted. One iv cannula was used for study drug administration and the cannula in the contralateral arm for PK sampling (Supplementary Methods). After administration, safety assessments (blood pressure, pulse, peripheral oxygen saturation and temperature) and blood collection for pharmacokinetics were performed regularly from just prior to administration up to the end of surgery. A stopping rule was defined in the protocol. In case of treatment-related serious adverse events or results suggesting futility to continue, the trial would be halted or stopped.

### Surgical procedure in ovarian cancer patients

All surgical procedures were performed by laparotomy through a midline abdominal incision. After opening of the abdominal cavity, the surgical field was searched for the primary tumor and metastases visible by the naked eye or palpation. Thereafter, the Artemis imaging system (see below for details) was used to identify fluorescent signals. When a fluorescent signal was observed, the operating surgeon performed a biopsy or resection of the fluorescent tissue. In case of non-fluorescence, only lesions macroscopically suspect for malignancy were resected. Resected specimens were marked as clinically suspect or not clinically suspect and as fluorescent or non-fluorescent. All resected lesions were examined by a pathologist for tumor status. In addition, an immunohistochemical (IHC) staining for FRα expression and fluorescence microscopy (Leica DM5500B fluorescence microscope) were performed to assess concordance of fluorescence with tumor and FRα presence and to evaluate binding sites of EC17 (Supplementary Methods).

To assess the number of malignant lesions that were identified by the naked eye and/or fluorescence imaging, stills from intraoperative obtained videos were analyzed by three dedicated gynecologic oncologists, experienced in ovarian cancer surgery. A total of 5 different images were analyzed. Each image was analyzed 3 times (normal, horizontally and vertically flipped), leading to a total of 15 images to be analyzed. First, only colour images were used to score the number of observed metastatic lesions. When this was completed, colour images supplemented with fluorescence images were scored.

### Surgical procedure in breast cancer

Patients underwent standard of care breast ablation or BCS both combined with SLN biopsy. The Artemis fluorescence imaging system was used to identify fluorescent signal during surgery and on resected specimens transferred to the pathology department. Intra-operative images of SLNs, the surgical field, resected specimen, and wound bed after resection were obtained. Following standard procedure, the resected specimen was dissected at the pathology department, where images from the dissected tumor were obtained as well. The resected specimens were routinely examined by a pathologist for tumor status. In addition, IHC staining for FRα expression and fluorescence microscopy (Leica DM5500B fluorescence microscope) were performed to evaluate binding site of EC17 (Supplementary Methods).

### Artemis fluorescence imaging system

Imaging procedures were performed using the Artemis fluorescence imaging system (Quest Medical Imaging, The Netherlands). The system consists of 3 wavelength isolated light sources: a “white” light source, and a two different “near-infrared” light sources. For this study, the camera and light engine were optimized for EC17 to generate 7.5 mW/cm^2^ at 490 nm light. Color video and fluorescence images are simultaneously acquired on separate sensors and displayed in real time using custom optics and software showing the separate the color video and NIR fluorescence images. A pseudo-colored (lime green) merged image of the color video and fluorescence images is also displayed. The intensities of the light sources could be controlled from the Artemis software. The camera can be attached to a freely moveable arm head. For intraoperative use, the camera and freely moveable arm were wrapped in a sterile shield and drape (Medical Technique Inc., Tucson, AZ).

### Statistical and image analysis

SPSS statistical software package (Version 20.0, Chicago, IL) was used. Patient characteristics were reported in median, standard deviation (SD), and range. PK parameters (AUC, Cmax, tmax) were statistically summarized including number of subjects, mean, standard deviation (SD), median, minimum and maximum. Plasma drug concentrations were plotted versus time per individual using both a linear and log y-axis. Additionally, concentration versus time curves were plotted as a spaghetti plot with the median added.

Fluorescent signal in tumor and background was quantified using ImageJ (version 1.49b; a public domain, Java-based image processing program developed at the National Institute of Health). Regions of interest (RoI) were drawn with ImageJ on the stored images to quantify fluorescent signal in arbitrary units [AU]. Tumor to background ratios (TBRs) were calculated by dividing the fluorescent signal of the tumor by fluorescent signal of surrounding tissue. To compare TBR and background signal between malignant and benign lesions independent samples t-test was used. TBR was reported in mean, SD, and range.

## SUPPLEMENTARY METHODS, FIGURE AND TABLE



## References

[1] Frangioni JV (2003). In vivo near-infrared fluorescence imaging. Curr.Opin.Chem.Biol.

[2] Weissleder R, Pittet MJ (2008). Imaging in the era of molecular oncology. Nature.

[3] Pleijhuis RG, Graafland M, de VJ, Bart J, de jong JS, van Dam GM (2009). Obtaining adequate surgical margins in breast-conserving therapy for patients with early-stage breast cancer: current modalities and future directions. Ann.Surg.Oncol.

[4] Chang SJ, Bristow RE, Ryu HS (2012). Impact of complete cytoreduction leaving no gross residual disease associated with radical cytoreductive surgical procedures on survival in advanced ovarian cancer. Ann.Surg.Oncol.

[5] Stummer W, Pichlmeier U, Meinel T, Wiestler OD, Zanella F, Reulen HJ (2006). Fluorescence-guided surgery with 5-aminolevulinic acid for resection of malignant glioma: a randomised controlled multicentre phase III trial. Lancet Oncol.

[6] Bristow RE, Berek JS (2006). Surgery for ovarian cancer: how to improve survival. Lancet.

[7] Bristow RE, Tomacruz RS, Armstrong DK, Trimble EL, Montz FJ (2002). Survival effect of maximal cytoreductive surgery for advanced ovarian carcinoma during the platinum era: a meta-analysis. J.Clin.Oncol.

[8] Vergote I, Trope CG, Amant F, Kristensen GB, Ehlen T, Johnson N, Verheijen RH, van der Burg ME, Lacave AJ, Panici PB, Kenter GG, Casado A, Mendiola C (2010). Neoadjuvant chemotherapy or primary surgery in stage IIIC or IV ovarian cancer. N.Engl.J.Med.

[9] Hoskins WJ, McGuire WP, Brady MF, Homesley HD, Creasman WT, Berman M, Ball H, Berek JS (1994). The effect of diameter of largest residual disease on survival after primary cytoreductive surgery in patients with suboptimal residual epithelial ovarian carcinoma. Am.J.Obstet.Gynecol.

[10] Handgraaf HJ, Verbeek FP, Tummers QR, Boogerd LS, van de Velde CJ, Vahrmeijer AL, Gaarenstroom KN (2014). Real-time near-infrared fluorescence guided surgery in gynecologic oncology: a review of the current state of the art. Gynecol.Oncol.

[11] Vahrmeijer AL, Hutteman M, van der Vorst JR, van de Velde CJ, Frangioni JV (2013). Image-guided cancer surgery using near-infrared fluorescence. Nat.Rev.Clin.Oncol.

[12] Patterson MS, Chance B, Wilson BC (1989). Time resolved reflectance and transmittance for the non-invasive measurement of tissue optical properties. Appl.Opt.

[13] Weissleder R, Ntziachristos V (2003). Shedding light onto live molecular targets. Nat.Med.

[14] Monici M (2005). Cell and tissue autofluorescence research and diagnostic applications. Biotechnol.Annu.Rev.

[15] van Dam GM, Themelis G, Crane LM, Harlaar NJ, Pleijhuis RG, Kelder W, Sarantopoulos A, de Jong JS, Arts HJ, van der Zee AG, Bart J, Low PS, Ntziachristos V (2011). Intraoperative tumor-specific fluorescence imaging in ovarian cancer by folate receptor-alpha targeting: first in-human results. Nat.Med.

[16] Burggraaf J, Kamerling IM, Gordon PB, Schrier L, de Kam ML, Kales AJ, Bendiksen R, Indrevoll B, Bjerke RM, Moestue SA, Yazdanfar S, Langers AM, Swaerd-Nordmo M (2015). Detection of colorectal polyps in humans using an intravenously administered fluorescent peptide targeted against c-Met. Nat.Med.

[17] Tummers QR, Verbeek FP, Schaafsma BE, Boonstra MC, van der Vorst JR, Liefers GJ, van de Velde CJ, Frangioni JV, Vahrmeijer AL (2014). Real-time intraoperative detection of breast cancer using near-infrared fluorescence imaging and Methylene Blue. Eur.J.Surg.Oncol.

[18] Tummers QR, Hoogstins CE, Peters AA, de Kroon CD, Trimbos JB, van de Velde CJ, Frangioni JV, Vahrmeijer AL, Gaarenstroom KN (2015). The Value of Intraoperative Near-Infrared Fluorescence Imaging Based on Enhanced Permeability and Retention of Indocyanine Green: Feasibility and False-Positives in Ovarian Cancer. PLoS.One.

[19] Maeda H, Wu J, Sawa T, Matsumura Y, Hori K (2000). Tumor vascular permeability and the EPR effect in macromolecular therapeutics: a review. J.Control Release.

[20] Matsumura Y, Maeda H (1986). A new concept for macromolecular therapeutics in cancer chemotherapy: mechanism of tumoritropic accumulation of proteins and the antitumor agent smancs. Cancer Res.

[21] low PS, Henne WA, Doorneweerd DD (2008). Discovery and development of folic-acid-based receptor targeting for imaging and therapy of cancer and inflammatory diseases. Acc.Chem.Res.

[22] Vergote IB, Marth C, Coleman RL (2015). Role of the folate receptor in ovarian cancer treatment: evidence, mechanism, and clinical implications. Cancer Metastasis Rev.

[23] Kalli KR, Oberg AL, Keeney GL, Christianson TJ, low PS, Knutson KL, Hartmann LC (2008). Folate receptor alpha as a tumor target in epithelial ovarian cancer. Gynecol.Oncol.

[24] O'Shannessy DJ, Somers EB, Smale R, Fu YS (2013). Expression of folate receptor-alpha (FRA) in gynecologic malignancies and its relationship to the tumor type. Int.J.Gynecol.Pathol.

[25] Parker N, Turk MJ, Westrick E, Lewis JD, low PS, Leamon CP (2005). Folate receptor expression in carcinomas and normal tissues determined by a quantitative radioligand binding assay. Anal.Biochem.

[26] Crane LM, Arts HJ, van OM, Low PS, van der Zee AG, van Dam GM, Bart J (2012). The effect of chemotherapy on expression of folate receptor-alpha in ovarian cancer. Cell Oncol.(Dordr.).

[27] Despierre E, Lambrechts S, Leunen K, Berteloot P, Neven P, Amant F, O'Shannessy DJ, Somers EB, Vergote I (2013). Folate receptor alpha (FRA) expression remains unchanged in epithelial ovarian and endometrial cancer after chemotherapy. Gynecol.Oncol.

[28] O'Shannessy DJ, Somers EB, Maltzman J, Smale R, Fu YS (2012). Folate receptor alpha (FRA) expression in breast cancer: identification of a new molecular subtype and association with triple negative disease. Springerplus.

[29] Liu TW, Stewart JM, Macdonald TD, Chen J, Clarke B, Shi J, Wilson BC, Neel BG, Zheng G (2013). Biologically-targeted detection of primary and micro-metastatic ovarian cancer. Theranostics.

[30] Vaitilingam B, Chelvam V, Kularatne SA, Poh S, Ayala-Lopez W, low PS (2012). A folate receptor-alpha-specific ligand that targets cancer tissue and not sites of inflammation. J.Nucl.Med.

[31] Kennedy MD, Jallad KN, Thompson DH, Ben-Amotz D, low PS (2003). Optical imaging of metastatic tumors using a folate-targeted fluorescent probe. J.Biomed.Opt.

[32] Altintas I, Kok RJ, Schiffelers RM (2012). Targeting epidermal growth factor receptor in tumors: from conventional monoclonal antibodies via heavy chain-only antibodies to nanobodies. Eur.J.Pharm.Sci.

[33] Choi HS, Gibbs SL, Lee JH, Kim SH, Ashitate Y, Liu F, Hyun H, Park G, Xie Y, Bae S, Henary M, Frangioni JV (2013). Targeted zwitterionic near-infrared fluorophores for improved optical imaging. Nat.Biotechnol.

[34] Hyun H, Park MH, Owens EA, Wada H, Henary M, Handgraaf HJ, Vahrmeijer AL, Frangioni JV, Choi HS (2015). Structure-inherent targeting of near-infrared fluorophores for parathyroid and thyroid gland imaging. Nat.Med.

[35] Oliveira S, Heukers R, Sornkom J, Kok RJ, van Bergen En Henegouwen PM (2013). Targeting tumors with nanobodies for cancer imaging and therapy. J.Control Release.

[36] Rosenthal EL, Warram JM, de BE, Chung TK, Korb ML, Brandwein-Gensler M, Strong TV, Schmalbach CE, Morlandt AB, Agarwal G, Hartman YE, Carroll WR, Richman JS (2015). Safety and Tumor Specificity of Cetuximab-IRDye800 for Surgical Navigation in Head and Neck Cancer. Clin.Cancer Res.

[37] Fader AN, Rose PG (2007). Role of surgery in ovarian carcinoma. J.Clin.Oncol.

[38] Bristow RE, Tomacruz RS, Armstrong DK, Trimble EL, Montz FJ (2002). Survival effect of maximal cytoreductive surgery for advanced ovarian carcinoma during the platinum era: a meta-analysis. J.Clin.Oncol.

[39] Hoskins WJ, McGuire WP, Brady MF, Homesley HD, Creasman WT, Berman M, Ball H, Berek JS (1994). The effect of diameter of largest residual disease on survival after primary cytoreductive surgery in patients with suboptimal residual epithelial ovarian carcinoma. Am.J.Obstet.Gynecol.

[40] Barranger E, Antomarchi J, Chamorey E, Cavrot C, Flipo B, Follana P, Peyrottes I, Chapellier C, Ferrero JM, Ihrai T (2015). Effect of Neoadjuvant Chemotherapy on the Surgical Treatment of Patients With Locally Advanced Breast Cancer Requiring Initial Mastectomy. Clin Breast Cancer.

[41] Soucy G, Belanger J, Leblanc G, Sideris L, Drolet P, Mitchell A, Leclerc YE, Dufresne MP, Beaudet J, Dube P (2008). Surgical margins in breast-conservation operations for invasive carcinoma: does neoadjuvant chemotherapy have an impact?. J.Am.Coll.Surg.

[42] Crane LM, Arts HJ, van OM, low PS, Van Der Zee AG, van Dam GM, Bart J (2012). The effect of chemotherapy on expression of folate receptor-alpha in ovarian cancer. Cell Oncol.(Dordr.).

[43] O'Shannessy DJ, Somers EB, Maltzman J, Smale R, Fu YS (2012). Folate receptor alpha (FRA) expression in breast cancer: identification of a new molecular subtype and association with triple negative disease. Springerplus.

[44] van Driel PB, van de Giessen M, Boonstra MC, Snoeks TJ, Keereweer S, Oliveira S, van de Velde CJ, Lelieveldt BP, Vahrmeijer AL, Lowik CW, Dijkstra J (2015). Characterization and evaluation of the artemis camera for fluorescence-guided cancer surgery. Mol.Imaging Biol.

[45] Zhang Z, Wang J, Tacha DE, Li P, Bremer RE, Chen H, Wei B, Xiao X, Da J, Skinner K, Hicks DG, Bu H, Tang P (2014). Folate receptor alpha associated with triple-negative breast cancer and poor prognosis. Arch.Pathol.Lab Med.

[46] Monici M, Basile V, Romano G, Evangelisti L, Lucarini L, Attanasio M, Bertini E, Fusi F, Gensini GF, Pepe G (2008). Fibroblast autofluorescence in connective tissue disorders: a future tool for clinical and differential diagnosis?. J.Biomed.Opt.

[47] Weissleder R, Ntziachristos V (2003). Shedding light onto live molecular targets. Nat.Med.

[48] De JE, Keating JJ, Kularatne SA, Jiang J, Judy R, Predina J, Nie S, Low P, Singhal S (2015). Comparison of Folate Receptor Targeted Optical Contrast Agents for Intraoperative Molecular Imaging. Int.J.Mol.Imaging.

[49] O'Shannessy DJ, Yu G, Smale R, Fu YS, Singhal S, Thiel RP, Somers EB, Vachani A (2012). Folate receptor alpha expression in lung cancer: diagnostic and prognostic significance. Oncotarget.

