# Supplementary material for: Intraoperative imaging of folate receptor alpha positive ovarian and breast cancer using the tumor specific agent EC17
Source: Oncotarget. 2016 Mar 22;7(22):32144–55. doi: 10.18632/oncotarget.8282 (PMC5078003; doi:10.18632/oncotarget.8282)
Supplement: Supplementary file 1 [file oncotarget-07-32144-s001.pdf]

Intraoperative imaging of folate receptor alpha positive ovarian and breast cancer using the tumor specific agent EC17

SUPPLEMENTARY METHODS

Immunohistochemistry

Immunohistochemical staining was performed on frozen sections of 5 µm. Sections were fixed in aceton for 10 minutes, dried and washed in PBS. Sections were stained using the Folate Receptor alpha IHC Assay Kit (Biocare Medical, BRI4006KAA). At first, nonspecific sites were blocked with a protein block for 5 minutes followed by incubation with Folate Receptor alpha antibody (or FRalpha Negative Control Reagent) for 30 minutes at room temperature. After a 10 minute incubation with MACH 4 Mouse Probe, sections were incubated with MACH 4 HRP Polymer. Finally, antibody binding was visualized by using 3,3'-diaminobenzidine. Sections were counterstained with haematoxylin, dehydrated and mounted with pertex.

Haematoxylin and eosin staining

Haematoxylin and eosin staining was performed on unfixed frozen sections. Sections were incubated in heamatoxylin for 5 minutes, washed in tap water for 5 minutes followed by incubation in eosin for 2 minutes. After thorough washing in demi water, sections were mounted with pertex.

Fluorescence microscopy

Frozen sections of 5 µm were mounted with Prolong Gold with DAPI (Life Technologies). Fluorescent signals were detected using a Leica DM5500B fluorescence microscope.

Pharmacokinetics

PK sampling was performed pre-dose, 15, 30, 45 and 60 min after administration of EC-17 and thereafter hourly until the end of surgery. Immediately following collection, the PK samples were put on ice and protected from light. Tubes were centrifuged at approximately 2000 g for 10 minutes at 2 to 8 °C. The separated plasma was transferred into labelled polypropylene tubes to avoid carry-over of erythrocytes. All samples were stored in an upright position at – 20 °C until analysis.

EC17 is extracted from buffered plasma by solid phase extraction using weak anion exchange cartridges. Chromatographic separation is performed on an Agilent Extend C18 column using gradient elution. An API 5500 tandem mass spectrometer equipped with a turbo spray ionisation source operating in MRM negative mode is used for data acquisition. The assay had a lower limit of quantification (LLOQ) and an upper limit of quantification (ULOQ) of 2.00 – 500 ng/ml.

Difference in autofluorescence signal at 500nm and 800nm

Difference in background fluorescence between 500nm and 800nm were measured in two patients undergoing standard of care breast cancer surgery in het LUMC. These patients were not included in a clinical trial and were not administered a fluorescence contrast agent. After resecting and dissecting the specimen, fluorescence imaging was performed using the described Artemis imaging system, optimized for 490nm, and another Artemis imaging system, optimized for 760nm.

Figure S1A shows fluorescence imaging at 500nm of breast tissue with high background signal, while Figure S1B shows fluorescence imaging at 800nm of the same patient. Here no fluorescent background signal is seen. The dashed circles indicate the location of the tumor.

SUPPLEMENTARY FIGURE AND TABLE

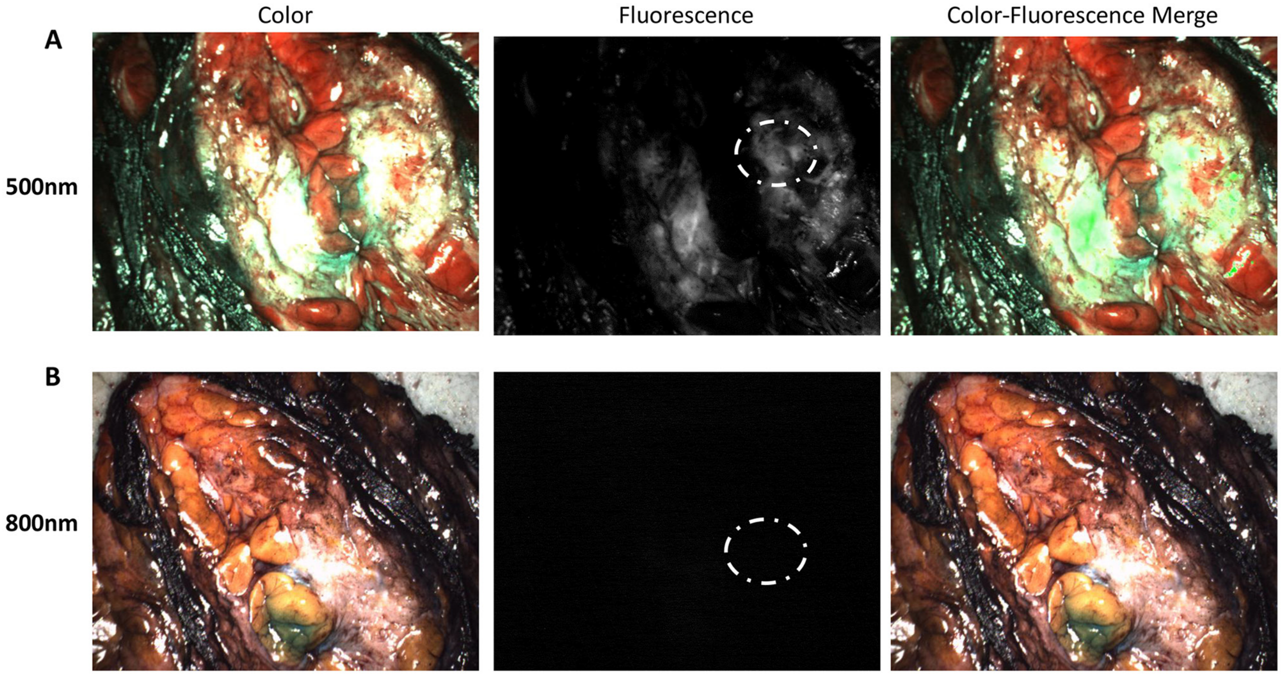

Supplementary Figure S1: Difference in autofluorescence signal at 500nm and 800nm. Fluorescence imaging of a disected breast cancer lesion (dashed circles) from a patient not treated with an exogenous contrast agent. **A.** shows the autofluorescence background signal at 500nm. Diffusely fluorescent background signal is observed. **B.** shows fluorescence imaging of the same lesion at 800nm. No fluorescent background signal is observed at this wavelength.

Supplementary Table S1: Summary of number of subjects with treatment emergent adverse events by SOC and preferred term and relationship

System Organ Class/ Preferred Term	0.1mg/kg EC17 (N=15)					
	Drug-related		Not (likely) drug-related		Total	
	N	%	N	%	N	%
ANY EVENTS	7	46.7	10	66.7	12	80
EYE DISORDERS	1	6.7	-	-	1	6.7
Dry eye	1	6.7	-	-	1	6.7
GASTROINTESTINAL DISORDERS	5	33.3	6	40.0	7	46.7
Abdominal discomfort	1	6.7	-	-	1	6.7
Abdominal pain	1	6.7	-	-	1	6.7
Constipation	-	-	1	6.7	1	6.7
Flatulence	-	-	1	6.7	1	6.7
Nausea	1	6.7	3	20.0	4	26.7
Reflux gastritis	-	-	1	6.7	1	6.7

(Continued)

System Organ Class/ Preferred Term	0.1mg/kg EC17 (N=15)					
	Drug-related		Not (likely) drug-related		Total	
	N	%	N	%	N	%
Throat irritation	1	6.7	-	-	1	6.7
Vomiting	1	6.7	-	-	1	6.7
GENERAL DISORDERS AND ADMINISTRATION SITE CONDITIONS	-	-	2	13.3	2	13.3
Fatigue	-	-	1	6.7	1	6.7
Oedema peripheral	-	-	1	6.7	1	6.7
IMMUNE SYSTEM DISORDERS	2	13.3	1	6.7	1	6.7
Pruritus allergic	2	13.3	1	6.7	1	6.7
INFECTIONS AND INFESTATIONS	-	-	2	13.3	2	13.3
Cystitis	-	-	1	6.7	1	6.7
Oral candidiasis	-	-	1	6.7	1	6.7
MUSCULOSKELETAL AND CONNECTIVE TISSUE DISORDERS	1	6.7	-	-	1	6.7
Sensation of heaviness	1	6.7	-	-	1	6.7
RENAL AND URINARY DISORDERS	-	-	1	6.7	1	6.7
Urinary incontinence	-	-	1	6.7	1	6.7
REPRODUCTIVE SYSTEM AND BREAST DISORDERS	-	-	1	6.7	1	6.7
Vaginal discharge	-	-	1	6.7	1	6.7
RESPIRATORY, THORACIC AND MEDIASTINAL DISORDERS	3	20.0	-	-	3	20.0
Sneezing	3	20.0	-	-	3	20.0
SKIN AND SUBCUTANEOUS TISSUE DISORDERS	1	6.7	7	46.7	8	53.3
Flushing	1	6.7	-	-	1	6.7
Pruritus generalised	-	-	1	6.7	1	6.7
Rash	-	-	1	6.7	1	6.7
Scar pain	-	-	5	33.3	5	33.3
VASCULAR DISORDERS	-	-	1	6.7	1	6.7
Lymphoedema	-	-	1	6.7	1	6.7
